# Combining Transcatheter Edge-to-Edge Repair and Commissural Plug Occlusion for Complex Degenerative Mitral Regurgitation

**DOI:** 10.1016/j.jaccas.2026.108350

**Published:** 2026-07-08

**Authors:** Yan-Jie Li, Lan Ma, Zi-Yong Hao, Xin Pan

**Affiliations:** a Department of Cardiology, Shanghai Chest Hospital, Shanghai Jiao Tong University School of Medicine, Shanghai, China; b Department of Ultrasonography, Shanghai Chest Hospital, Shanghai Jiao Tong University School of Medicine, Shanghai, China

**Keywords:** chronic heart failure, echocardiography, image, mitral valve, murmur, valve repair

## Abstract

**Background:**

Degenerative mitral regurgitation involving the commissures remains a challenging anatomical subset for transcatheter repair.

**Case Summary:**

An 86-year-old male with high surgical risk presented with symptomatic, severe degenerative mitral regurgitation due to A3 prolapse extending to the posteromedial commissure and a measured mitral valve area of 3.8 cm^2^. The heart team was elected for transcatheter edge-to-edge repair. Despite successful implantation of a single clip in the A3-P3 region, moderate residual commissural regurgitation persisted. The small native valve area and insufficient leaflet length after the first clip precluded safe placement of a second device. As a bailout strategy, a vascular plug was deployed to specifically occlude the residual commissural leak.

**Discussion:**

This simultaneous hybrid approach successfully reduced mitral regurgitation to mild, demonstrating a novel solution for managing complex anatomical challenges where conventional transcatheter edge-to-edge repair is insufficient.

**Take-Home Messages:**

Commissural involvement combined with a small mitral valve area and limited leaflet length may render conventional multiclip transcatheter edge-to-edge repair unfeasible, increasing the risk of residual regurgitation. This hybrid transcatheter strategy, combining edge-to-edge repair with targeted plug occlusion, can expand the treatable spectrum of complex degenerative mitral regurgitation in high-surgical-risk patients.

## Visual Summary

**Figure:**
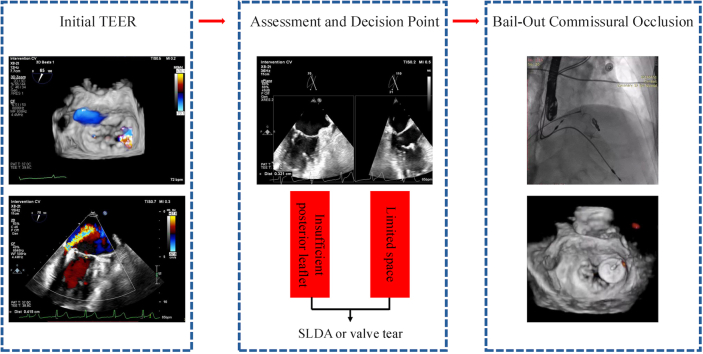

**Visual Summary:**
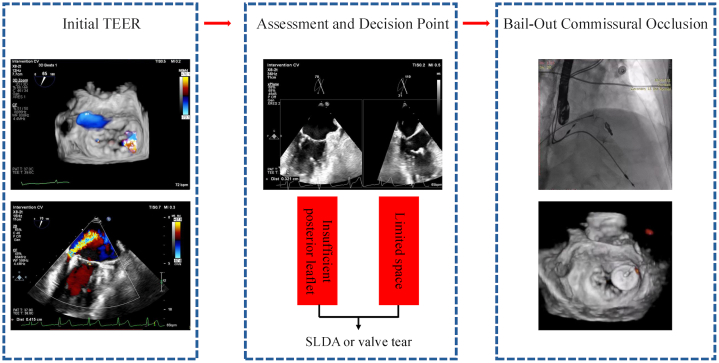
Hybrid Strategy Combining Mitral Transcatheter Edge-to-Edge Repair and Commissural Plug Occlusion for Residual Regurgitation

While transcatheter edge-to-edge repair (TEER) is a well-established therapy for degenerative mitral regurgitation (DMR),^1^ complex anatomies, such as commissural involvement, small annular dimensions, and limited leaflet tissue, pose significant challenges. Suboptimal results with residual leaks are common in these scenarios, necessitating innovative bailout strategies. We present a case where a hybrid approach, combining TEER with simultaneously targeted commissural occlusion using vascular plug, effectively addressed a complex DMR pathology.

**Take-Home Messages**

• Commissural involvement combined with a small mitral valve area and limited leaflet length may render conventional multiclip transcatheter edge-to-edge repair unfeasible, increasing the risk of residual regurgitation.
• This hybrid transcatheter strategy, combining edge-to-edge repair with targeted plug occlusion, can expand the treatable spectrum of complex degenerative mitral regurgitation in high-surgical-risk patients.

## History of Presentation

An 86-year-old man with hypertension and paroxysmal atrial fibrillation presented with progressive fatigue and dyspnea on exertion (New York Heart Association functional class III). Physical examination revealed a holosystolic murmur at the apex and signs of mild volume overload.

## Past Medical History

The patient underwent dual-chamber pacemaker implantation due to sick sinus syndrome 10 years ago and cataract surgery. He had a history of benign prostatic hyperplasia without diabetes mellitus or stroke.

## Differential Diagnosis

The differential diagnosis for this patient's presentation included the following conditions: functional mitral regurgitation (MR), acute chordal rupture, mitral annular calcification with associated regurgitation, aortic valve disease, and nonvalvular causes of dyspnea.

## Investigation

**Figure 1:**
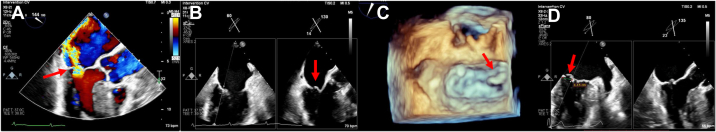
Diagnostic Evaluation (A to C) Severe mitral regurgitation originating from a flail A3 scallop with extension into the posteromedial commissure. (D) The width of the prolapse.

Laboratory values showed an elevated N-terminal pro–B-type natriuretic peptide level of 2,300 ng/L. Transthoracic echocardiogram demonstrated normal left ventricular systolic function with ejection fraction of 58%, enlarged left ventricular end-diastolic diameter of 63 mm, and severe eccentric anteriorly directed MR. It also showed normal right ventricular size and function and an estimated pulmonary artery systolic pressure of 48 mm Hg. Transesophageal echocardiography (TEE) revealed severe complex MR along posteromedial commissure with significant jet originating from a flail A3 scallop with extension to the center (Figures 1A to 1C, Videos 1 and 2), and the width of prolapse is 11.5 mm (Figure 1D). The mitral valve area by planimetry was 3.8 cm^2^. Coronary angiography and right heart catheterization demonstrated no obstructive coronary artery disease, right atrial pressure of 8 mm Hg, pulmonary artery pressure of 41/18 (26) mm Hg, and pulmonary capillary wedge pressure of 18 mm Hg. Fick cardiac index was 2.6 L/min/m^2^. Given the patient's advanced age, symptomatic status, and noteworthy surgery risk scores (Society of Thoracic Surgeons score for mitral valve surgery: 9.5%), the heart team opted for a percutaneous intervention.

## Management

The plan was to treat the DMR proceed with mitral TEER using MitraClip G4 (Abbott). The procedure was performed under general anesthesia with fluoroscopic and real-time 3D TEE guidance.

**Figure 2:**
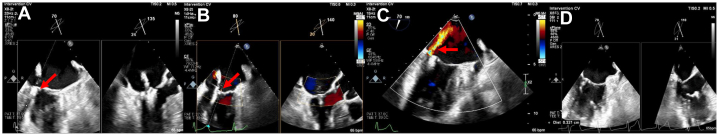
Initial Mitral Transcatheter Edge-to-Edge Repair (A) The MitraClip positioned at the A3-P3 and posteromedial commissure. (B) The MitraClip deployed in the A3-P3. (C) A moderate residual jet from the commissural aspect. (D) The length of posterior leaflet medial to the clip.

Given the large width of mitral valve prolapse and the small mitral valve orifice area, a single-clip strategy was selected. A MitraClip NTW was positioned more laterally to treat the jet at A3-P3 and posteromedial commissure, as initially planned (Figure 2A). However, residual regurgitant jet was observed on both sides of the implanted clip, with the greater portion located laterally, closer to the A2-P2 segment. In addition, the mean transmitral gradient elevated to 5 mm Hg, indicating the risk of iatrogenic stenosis with another clip. Then, the MitraClip was moved slightly to adjust the position and finally deployed in the A3-P3 region under TEE guidance (Figure 2B), grasping the whole flail segment, which reduced the MR but left a moderate residual jet, originating from the commissural aspect (Figure 2C).

Detailed TEE assessment postclip revealed the anatomical constraints of the patient: the risk of entanglement was judged elevated if a new clip was attempted to deploy in the limited space, and if the posterior leaflet length (3.2 mm) (Figure 2D) adjacent to the first clip was insufficient for stable and safe capture by a second clip, which can result in single leaflet device attachment or valve tear. Thus, the conventional multiclip strategy was deemed unfeasible.

**Figure 3:**
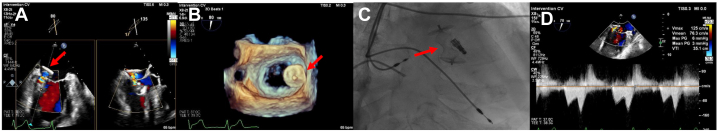
Bail-Out Commissural Occlusion and Final Result (A to C) Amplatzer Vascular Plug device at the A3-P3 position and mild residual commissural regurgitation. (D) The final mean transmitral gradient.

To address the specific, localized regurgitant orifice, the team opted for a device-based occlusion. First, the regurgitant orifice dimensions were carefully sized by 3-dimensional TEE imaging and measured at approximately 10 × 6 mm. For the medial jet with adjunctive closure of the lateral, an 18-mm Amplatzer Vascular Plug (AVP, Abbott Vascular) II was selected. The MitraClip guide catheter was then exchanged for a 6-F Judkins right 4 diagnostic catheter, which was placed in the left atrium. Under 3-dimensional TEE guidance and fluoroscopy, the medial commissure was crossed using a glide wire. An 8-F March 1 guiding catheter (Boston Scientific) was subsequently exchanged, which was placed in the left ventricle through the gap. Afterward, the AVP II was advanced through the guiding catheter without any resistant. Finally, the AVP device was located directly between the medial commissure and the clip for the regurgitant jet origin. Subsequent TEE imaging demonstrated a stable device position, and the left ventricular disc was ensured full coverage without protrusion into the left ventricular inflow tract and increasing gradient, which resulted in immediate color Doppler and hemodynamic improvement with trace to mild regurgitation flow through the AVP device at the A3-P3 position and reduction in the degree of residual commissural MR (Figures 3A to 3C, Videos 3 and 4). The mean left atrial pressure decreased to 9 mm Hg. The mean transmitral gradient was 3 mm Hg (Figure 3D), and the valve area was preserved to 3.0 cm^2^.

## Follow-Up

The patient experienced immediate symptomatic relief without hemolysis and was maintained on anticoagulant therapy due to atrial fibrillation. An electrocardiogram obtained on the day of the intervention demonstrated left anterior fascicular block, which normalized by the following day. Early postprocedural imaging demonstrated mild residual transplug jets, which progressively decreased at follow-up. Three-month postprocedural transthoracic echocardiography confirmed stable device position, mild residual MR, mean transmitral gradient of 4 mm Hg, and absence of thrombotic material.

## Discussion

The use of vascular or septal occluder devices as a bailout strategy after suboptimal TEER has been reported in a growing number of case reports and small series.^2^^,^^3^ The primary advantage of the strategy is that it provides a rescue option when conventional TEER fails to achieve optimal MR reduction, but a second clip cannot be safely placed. The vascular plug, with its low-profile delivery system and ability to occlude a discrete orifice without requiring leaflet grasping, offered a viable alternative. The second advantage is preservation of the mitral valve area. By using a plug rather than a second clip, the hybrid approach seals the residual defect without adding to the leaflet coaptation area. The third advantage is precision targeting of residual orifice. The residual regurgitation after the initial TEER in commissural pathology is often a specific, localized defect. The plug-based approach successfully treated distinct types of residual leaks (leaflet tears, paraclip leaks, interclip, and leaks). Finally, this bailout can be performed in the same catheterization laboratory session, avoiding conversion to surgical repair in a high-risk patient. However, the most concerning data come from Maisano's group.^4^ Despite satisfactory procedural results, an overall 30-day mortality was 38%, and procedural success at 30 days was only 38%, which raises serious questions about the safety of this approach. Hemolysis has been reported as a complication of plug-based mitral valve interventions, likely due to incomplete endothelialization or persistent high-velocity flow through residual small channels. In addition, device embolization remains a potential catastrophic complication. Finally, perhaps the most significant disadvantage is the absence of systematic long-term follow-up data. The plug is not anchored in the same manner as a clip; its stability relies on radial force within the regurgitant orifice, which may change over time as valve morphology evolves or as left ventricular remodeling occurs.

In our rare experience, plug occlusion is suitable when the regurgitant jet is relative narrow and deep like tunnel with a defined waist. However, device occlusion unfits when anchoring struts are absent, or when the device would risk interfering with leaflet motion or producing a prohibitive transmitral gradient. The optimal device type and size were chosen according to TEE findings. Fluoroscopic assessment is useful for catheter crossing, occluder location, and “push and pull” test. The gap width between the commissural annulus and clip can be estimated by 3-dimensional quantification measurement based on the maximum width and residual regurgitant jet. On TEE imaging, 3-dimensional quantification measurement of the residual regurgitant orifice is helpful to select optimal device size. Thus, commissural/paracommissural locations may be feasible in highly selected anatomies but demand meticulous imaging, cautious sizing, and clear bailout strategy.

The novelty of this case lies in the hybrid transcatheter strategy simultaneously combining TEER and a vascular plug to address a residual commissural leak when conventional multiclip repair was anatomically infeasible. The case introduces a reproducible, anatomy-driven rescue maneuver for a challenging clinical scenario that lacks established guidelines, potentially changing the paradigm for managing residual commissural MR after TEER. While careful hemodynamic monitoring remains essential to guide intraprocedural decision-making and avoid functional mitral and left ventricular inflow stenosis. In addition, long-term durability, risk of thrombosis, and effects on leaflet dynamics require further evaluation. This case adds to the growing armamentarium for managing challenging DMR and suggests a potential niche for combined device therapies.

## Conclusions

For high-surgical-risk patients with complex DMR involving the commissure and restrictive anatomy, the hybrid approach, primary TEER followed by targeted commissural plug occlusion for a residual leak, can be a viable and effective rescue strategy. It exemplifies adaptive, patient-specific problem-solving in the catheterization laboratory.

## Funding Support and Author Disclosures

This study was supported by grants from the Shanghai Committee of Science and Technology, China (24SF1904804, 24SF1902004, and 25SF1902404). The authors have reported that they have no relationships relevant to the contents of this paper to disclose.

## Appendix

Video 1 Mitral Regurgitation Along the Anterior-Lateral Commissure With Significant Jet Originating From a Flail A3 Scallop on Transesophageal Echocardiography

Video 2 Degenerative Mitral Regurgitation With a Flail A3 Scallop on Transesophageal Echocardiography

Video 3 Final Echocardiography of Mitral Regurgitation

Video 4 Final Fluoroscopy of the Clip and Plug
